# Impact of Unexpected Death in a Simulation Scenario on Skill Retention, Stress, and Emotions: A Simulation-Based Randomized Controlled Trial

**DOI:** 10.7759/cureus.39715

**Published:** 2023-05-30

**Authors:** Kristina Khanduja, M. Dylan Bould, Meghan Andrews, Vicki LeBlanc, Karl Schebesta, Joseph K Burns, Richard Waldolf, Pratheeban Nambyiah, Jennifer Dale-Tam, Charles Henri Houzé-Cerfon, Sylvain Boet

**Affiliations:** 1 Department of Anesthesiology, Mount Sinai Hospital/University of Toronto, Toronto, CAN; 2 Department of Innovation in Medical Education, University of Ottowa, Ottowa, CAN; 3 Clinical Epidemiology Program, Ottawa Hospital Research Institute, Ottawa, CAN; 4 Department of Anesthesiology and Pain Medicine, Montfort Hospital/University of Ottawa, Ottawa, CAN; 5 Department of Innovation in Medical Education, University of Ottawa, Ottawa, CAN; 6 Department of Anesthesia, Intensive Care Medicine and Pain Medicine, Medical University of Vienna, Vienna, AUT; 7 Department of Anesthesiology and Pain Medicine, The Ottawa Hospital/University of Ottawa, Ottawa, CAN; 8 Department of Family Medicine, University of Ottawa, Ottawa, CAN; 9 Department of Anaesthesia, Great Ormond Street Hospital for Children, London, GBR; 10 Nursing Simulation Education, The Ottawa Hospital/University of Ottawa, Ottawa, CAN; 11 Department of Emergency Medicine, Toulouse University Hospital, Toulouse, FRA

**Keywords:** simulation-based education, skill retention, non-technical skills, crisis resource management skills, simulated patient death

## Abstract

Introduction

The simulation of patient death remains controversial in simulation-based education. We investigated the effect of simulated patient death on learners' skill retention, stress levels, and emotions.

Methods

After ethics approval, we recruited residents at two Canadian universities. Participants were randomized to manage a simulated cardiac arrest ending with either the unexpected death (intervention group) or survival (control group) of the simulated patient (i.e., manikin). Three months later, all participants performed the same scenario but with the opposite outcome. Blinded video raters assessed participants' non-technical and technical crisis resource management (CRM) skills at both time points. Stress levels (represented by anxiety level, salivary cortisol concentration, and cognitive appraisal) and emotional valence were measured. Outcomes were analyzed using analysis of covariance (ANCOVA) or generalized estimating equations as appropriate.

Results

The analysis included 46 participants (intervention: n=24; control: n=22). Simulated death neither affected retention of non-technical CRM skills (mean retention Ottawa Global Rating Scale score in the death group [29.4, 95% CI: 27.0, 31.8] versus control group [29.4, 95% CI: 26.8, 32.0; p*=*0.87]) nor technical CRM skills (mean retention task-specific checklist score in the manikin death group [11.8, 95% CI: 10.5, 13.0] versus the control group [12.5, 95% CI: 11.3, 13.7; p=0.69]). The simulated death had negative effects on participants' anxiety levels, cognitive appraisal, and emotions.

Conclusion

Simulated patient death did not affect the retention of non-technical or technical CRM skills but led to greater levels of short-term anxiety, stress, and negative emotions among learners.

## Introduction

The sudden and unexpected death of a patient is one of the most feared outcomes in clinical practice and has considerable psychological effects on healthcare workers. In simulation-based education, the educator is able to decide whether death is simulated or not, resulting in the dilemma of whether patient death should be used in simulation education.

Although patient death is common after in-hospital cardiac arrest [1], educators are often reluctant to incorporate death into simulation scenarios. Simulated patient death describes a situation in which death is not the primary learning objective [2,3]. The occurrence of unexpected death during simulation is highly controversial: little data is available to inform its effect on learning [2,4,5]. Some educators argue that incorporating death in the safe setting of a simulation environment may prepare learners for clinical practice [4] or may be necessary to maintain model authenticity if management decisions cause the simulated situation to deteriorate to the point where no other outcome is realistic. Others consider that the occurrence of simulated patient death may create an emotional "to-be-remembered event" with the potential for enhanced memory consolidation [6]. Other educators, however, contend that learners may view an unexpected death as "punishment," that it may exacerbate stress and negative emotions, that it may divert attention from the learning objective, and that it may ultimately cause learners to refuse to participate in future simulation sessions [2,5,7].

The impact of simulated death on participants' learning has been previously discussed in opinion papers and retrospective surveys expressing divided opinions, sometimes at extremes [2,4,8]. Stress results from the perception that one doesn't have the resources to meet the perceived demands of a situation. This can lead to negative emotional states (e.g., anxiety, anger) and specific physiological responses (e.g., elevations in salivary cortisol) that, in turn, can influence cognitive processes critical to learning, such as attention and memory. Only recently has this question begun to be addressed using randomized controlled trials (RCTs). Two recent RCTs exploring the effect of simulated patient death on the retention of skills and various outcomes returned conflicting results [6,9]. Fraser et al. found that simulated patient death leads to poorer learning outcomes at three months and seems to be associated with higher cognitive load [9]. DeMaria et al. found no impact of simulated patient death on learning outcomes at six months and no impact on physiological stress markers [6]. In addition to conflicting findings, both of these previous studies had significant limitations in terms of using entirely novice participants (medical students) without robust measures of stress. One of these studies had a very small sample size, while the other used an unvalidated primary outcome measure. We believe that assessing the impact of simulated patient death requires (i) recruiting clinicians, such as post-graduate trainees or attendings, who likely have greater experience with these types of events (patient death, resuscitations), have greater clinical experience and knowledge to bring to the situation, and may have a better understanding that death is sometimes an unavoidable outcome despite correct clinical management, and (ii) selecting outcome measures of clinical performance and stress with stronger validity evidence.

We elected to explore the impact of simulated patient death in order to provide a comprehensive picture and offer a better understanding of the possible relationship among all the outcomes. We explored retention performance at three months, physiological and psychological stress markers [6,10-12], and emotional valence [13] after a simulated patient death. Our study aimed to assess the impact of simulated patient death (using a simulation manikin) on crisis resource management (CRM) skill retention, participants' stress levels, and emotions. Our primary objective was to evaluate the impact of a simulated patient death on crisis management skill retention at three months. We hypothesized that simulated patient death during simulated cardiac arrest would impair learning and be detrimental to participants' stress levels and emotions.

## Materials and methods

Study design

This study was a multicenter, prospective, double-blinded (participant and rater), randomized controlled trial (Table 1) and was registered on ClinicalTrials.gov (#NCT03441425). Following Research Ethics Board approval (Mount Sinai Hospital, University of Toronto, REB #14-0086-E and Ottawa Health Science Network REB #20130171-01H, Ontario, Canada), 56 residents from all medical specialties from two Canadian universities were invited to participate. Informed consent used deception to maintain participant blinding. Candidates who were pregnant or at risk of hypothalamic-pituitary-adrenal axis suppression were excluded. The study was reported following the Consolidated Standards of Reporting Trials (CONSORT) guideline [14] and its expansion for simulation studies [15].

**Table 1:** Overview of outcomes collected at different time points

Outcomes		Initial simulation session					Retention simulation scenario (three months)				
		Arrival at the simulation center	Before scenario	During scenario	Just after scenario	Just after debriefing	Arrival at the simulation center	Before scenario	During scenario	Just after scenario	Just after debriefing
Crisis management performance	Ottawa Global Rating Scale			X					X		
	Task-specific checklist			X					X		
Stress	Salivary cortisol	X	X		X	X X	X	X		X	X X
	State-Trait Anxiety Inventory		X		X	X		X		X	X
	Cognitive appraisal		X		X			X		X	
Emotions	Positive and negative affect schedule					X					X

Simulation scenarios and study conduct

Subjects were randomized to either the intervention group (simulated patient death) or the control group (simulated patient survival). In order to create groups that were similar in their baseline characteristics and increase the power of our study, randomization was stratified according to the study site, level of training (postgraduate years 1-3 vs. postgraduate years 4-5 and clinical fellows), and acute (anesthesiology, critical care, and emergency medicine) versus non-acute care (family medicine, obstetrics and gynecology [OB/GYN] and maxillofacial surgery) specialties. Computer randomization was performed using the algorithm provided at www.randomization.com.

All subjects participated in two 13-minute, manikin-based simulated in-hospital cardiac arrest scenarios as the code team leader with two embedded actors (Appendix A). Participants were blinded to their group assignment and corresponding scenario ending. The embedded actors received extensive training with pre-scripted responses and interactions and played the roles of a nurse and a respiratory therapist. Embedded actors were allowed to answer orders and questions from the participants but had to adhere to their scripted roles. For the simulated patient death group, the scenario ended with asystole as the final rhythm, regardless of the participant's performance. For the simulated patient survival group, the scenario ended with a return of spontaneous circulation (ROSC). A standardized, video-assisted debriefing was conducted without unblinding participants by the same debriefer at each site. Participants completed a retention test three months after the initial test.

In order to minimize recall bias, we used two different pre-brief stories (either stem 1 or stem 2) and two different sequences of cardiac rhythm (either pulseless electrical activity [PEA] followed by ventricular fibrillation [VF] or vice-versa) between the initial and the retention scenarios. For example, a participant from the simulated patient death group who managed a patient with background information from stem 1 and presenting with VF followed by PEA in the initial scenario had to manage a patient with background information from stem 2 presenting with PEA followed by VF in the retention test. Because that participant was in the simulated patient death group, the initial scenario ended by asystole, while the retention scenario ended by a ROSC. We chose to do so in order to increase the data collection for our secondary outcomes. All participants were once again debriefed following the final simulation scenario. In summary, the scenarios are of equal complexity but differ primarily with respect to the clinical context, initial cardiac arrest rhythm, and pre-determined outcome. Details are provided in Appendix A. Allocation of the stem and the sequence of cardiac rhythm were randomized at the time when subjects were randomized to either the intervention group (simulated patient death) or the control group (simulated patient survival).

Data collection

Participant Characteristics

Participant characteristics, including age, sex, post-graduate-year level, specialty, previous advanced cardiac arrest experience, and training, as well as simulation experience, were collected during the initial and retention phases.

Performance Assessment

Global rating scale: For the primary outcome, CRM skills were evaluated using the Ottawa Global Rating Scale (OGRS) [16,17]. After a rater training session, pairs of blinded, independent expert raters (RW and CHHC; JDT and PN) reviewed recorded scenarios. The OGRS is an assessment tool with strong validity evidence for the measurement of CRM skills, composed of five elements identified in the literature: situational awareness, leadership, communication, problem-solving, and resource utilization [17]. Raters use a seven-point Likert scale with anchored descriptors to assign a score for each of these five categories in addition to an overall score, resulting in a total score between six and 42 points [17].

Task-specific checklist (CL): As a secondary outcome, performance was also measured by using a task-specific checklist (CL) adapted from the American Heart Association's Megacode Checklist. The tool was modified with an intentional omission of post-arrest care as our reviewers were blinded to the scenario's ending. This assessment tool is composed of 18 specific tasks: raters assign a score of 1 if the task is performed according to the description and 0 if it is not. This results in a total score ranging between 0 and 18 points.

Stress Assessment

Acute stress was assessed by means of salivary cortisol concentration, the State-Trait Anxiety Inventory (STAI), and a cognitive questionnaire [10,18-20].

Salivary cortisol: Salivary cortisol levels correspond well to plasma cortisol levels and were measured at pre-determined time points as a surrogate marker of stress [18,19,21]. Salivary cortisol peaks 20 to 40 minutes after the onset of a stressor [18]. All participants were asked to refrain from physical strain, smoking, drinking caffeinated or low-pH beverages, and eating for at least one hour prior to the conduct of the study to ensure accuracy. In order to replicate cortisol kinetics, five cortisol samples were obtained per participant and per scenario: when participants arrived at the simulation center (sample 0), just before each scenario (sample 1), immediately at the end of the scenario (sample 2), 30 minutes after the start of the scenario (sample 3), and at the end of the debriefing phase (sample 4). Samples were collected by having participants chew on roll-shaped synthetic saliva collectors (Salivette for Cortisol testing, Starstedt, Montreal, Quebec, Canada) and then frozen at -20°C until analysis using an enzyme-linked immunosorbent assay (ELISA) technique at the Technische Universitat Dresden, Germany.

State-Trait Anxiety Inventory (STAI): The "state" section of the STAI questionnaire uses 20 statements for which participants select a score based on a four-point Likert-like scale (1 - not at all, 4 - very much so) to reflect changes in stress and anxiety, with a possible range of scores of 20 (least anxious) to 80 (most anxious) [10,20]. STAI state score was assessed at three pre-determined time points: prior to the scenario, following the scenario, and immediately after the debriefing on both the initial and retention simulation days.

Cognitive Appraisal

The cognitive appraisal was measured after the scenario pre-briefing and immediately after the scenario on both simulation days using the method originally described by Tomaka and colleagues [22,23], based on a model by Lazarus and Folkman [24] and previously modified and validated for use in simulation by Harvey and colleagues [10].

Immediately before the scenario, a primary appraisal (evaluation of perceived "demand" on the participant) was measured by asking the question, "How demanding do you expect the upcoming task to be?". Secondary appraisal (evaluation of perceived "resources" by the participant, including their own capacity) was assessed by asking, "How able are you to cope with this task?" After the scenario was completed, a further primary appraisal was conducted by asking, "How demanding was the task you just completed?", followed by a secondary appraisal using the question "How able were you to cope with this task?" All of these questions were answered using an anchored 10-item Likert scale.

A cognitive appraisal ratio was calculated thereafter by dividing the results of the primary appraisal by the results of the secondary appraisal. A ratio >1 indicates that resources do not meet demands and the task is appraised as a "threat". A ratio <1, where resources are greater than demands, will indicate the task is perceived as a "challenge."

Emotion Assessment

The death of the simulated patient can trigger a plethora of emotions [2]. A Positive and Negative Affect Schedule (PANAS) assessment was performed following the debrief [13]. The PANAS has strong validity evidence and presents participants with 20 emotions and asks them to use a five-point Likert scale to indicate how strongly they feel each emotion, ranging from 1 ("very slightly or not at all") to 5 ("extremely"). Participants self-reported their emotions in the present moment at the end of each simulation day using the PANAS. The positive affect (PA) items were separated from the negative affect (NA) items during analysis [13], as they are related but not additive.

Outcomes

Our primary outcome was the difference in retention of CRM skills (measured by the Ottawa Global Rating Scale), defined as the difference in performance between the initial and the retention tests between the intervention and control groups.

All secondary outcomes were considered exploratory and assessed the difference between the initial and the retention tests and between the intervention and control groups for a number of outcomes: (i) task-specific CL, (ii) stress markers, including salivary cortisol, cognitive appraisal and STAI, and (iii) valence of emotions.

Sample size calculation

Using the recommended sample size estimation approach for analysis of covariance (ANCOVA) [25] with an estimated r^2^ of 0.12 derived during a previous trial, we determined that a total of 46 participants would be needed. In order to account for an estimated attrition rate of 20%, we needed to recruit 56 participants. G*Power (version 3.12, Düsseldorf, Germany) was used for sample size calculations.

Statistical analysis

We established the inter-rater reliability of video scores provided by the four raters by calculating the two-way random intra-class correlation coefficient (ICC) for absolute agreement. Demographic data was analyzed using a chi-square test or Mann-Whitney U test, where appropriate. In line with recommendations for stratified randomized controlled trials, ANCOVA was used to analyze all outcomes, as appropriate, and used group assignment (simulated patient death or survival), the level of training (PGY 1-3 vs. PGY 4-5 and fellows), and specialty (acute care specialties vs. non-acute care specialties) as between-subjects independent variables [26]. To analyze the primary outcome of crisis management skills, we used the initial OGRS score as the covariate in the ANCOVA. For the secondary outcome of performance on the CL, we used the initial CL score as the covariate in a similar ANCOVA. Because the recordings ended before the participant experienced the simulated patient death (or survival) in each scenario, there was no crossover effect for these outcomes, as the scenario experienced on the retention test would not affect the participant's performance. Conversely, the collection of data for other secondary outcomes (cortisol levels, STAI, cognitive appraisal, and emotional valence) continued after the end of the scenario, and, therefore, these data were affected by the type of scenario experienced on both days. This created a crossover effect which necessitated analysis of these outcomes using generalized estimating equations (GEEs).

Cognitive appraisal results were analyzed using one-sample t-tests to determine whether the average ratio was different from 1. Wilcoxon signed rank tests were then used to compare the ratios of the two groups at each time point, as the data were not normally distributed. To analyze scores on the STAI, a GEE was used with the baseline scores used as the covariate and the day of the test (initial or retention) and the scenario (simulated patient death or survival) as factors. The test phase (i.e., the day of the test) was used as a within-subjects variable. Considering each participant completed both scenarios over the course of the study (one on the initial day and the other on the retention day), paired t-tests were subsequently used to compare the two scenarios in terms of the mean change in STAI score versus baseline at each of the remaining two-time points (after scenario and after debriefing). Cortisol concentrations were analyzed using a similar GEE with the baseline concentrations as the covariate. Changes in cortisol concentration over time within each group were analyzed using Friedman tests, as the concentrations were not normally distributed.

The positive scale and the negative scale of the PANAS were subjected to a GEE but without a baseline covariate, as this scale was measured only once on each study day. The scores on the negative scale were not normally distributed according to Shapiro-Wilk tests and were analyzed using Wilcoxon signed-rank tests. In all analyses, Likert-like scales were treated as interval measures and analyzed using parametric tests when the assumption of normality was not violated [27,28]. All secondary outcomes, including multiple comparisons, were considered exploratory.

## Results

**Figure 1:**
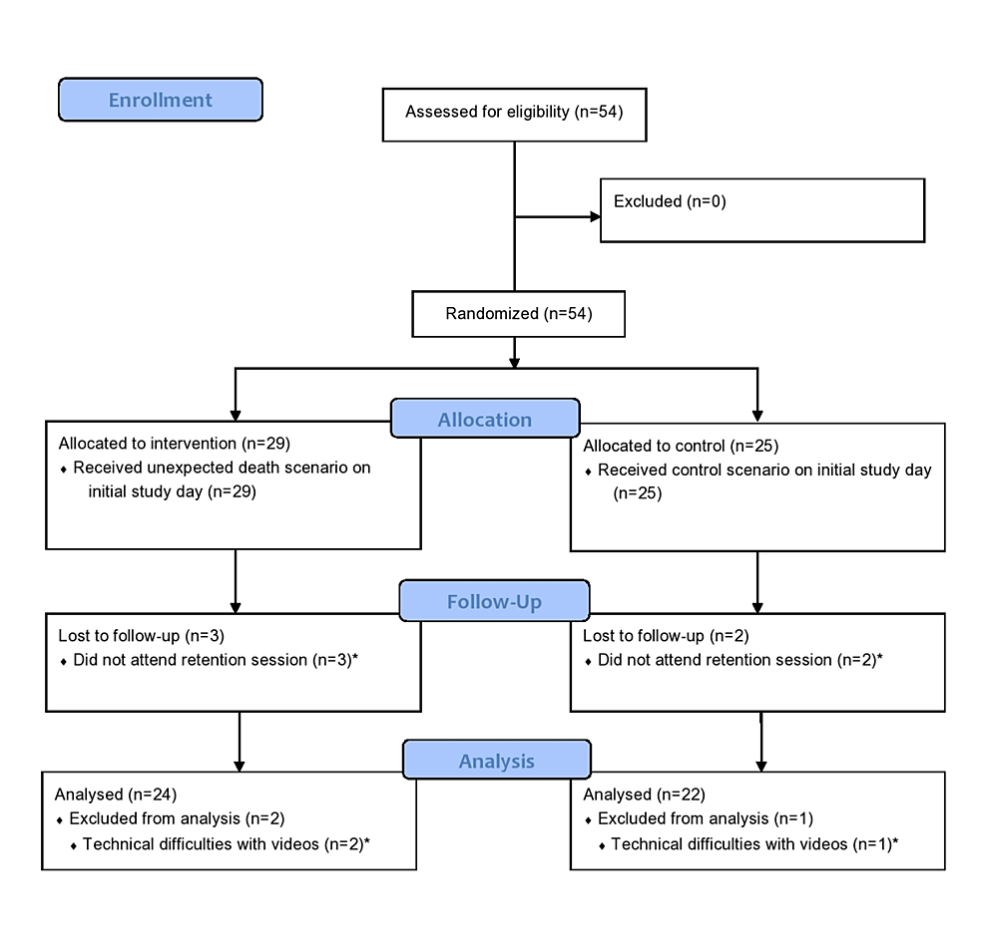
CONSORT flow diagram CONSORT - Consolidated Standards of Reporting Trials

Fifty-four participants were enrolled in the study and included in the analysis; five participants were unable to attend the retention test. Therefore, the analysis included 46 participants for the primary outcome and 54 for the secondary outcomes, as data were obtained from their initial tests (Figure 1).

Participant demographics are reported in Table 2. Recruitment began on November 10, 2014, and the final retention test occurred on September 29, 2017.

**Table 2:** Participant characteristics ACLS - advanced cardiovascular life support; ERT - emergency response team

		Death group (%)	Survival group (%)
Gender	Female	15 (52)	13 (52)
Level	Junior	21 (72)	19 (76)
Specialty	Acute	25 (86)	24 (96)
Member of ERT	Yes	12 (41)	14 (58)
ACLS certified	Yes	27 (93)	24 (96)
ACLS trainer	Yes	4 (14)	3 (12)

Inter-rater reliability

The overall inter-rater reliability for video scores was substantial: ICC=0.96 (p<0.001). Considering the high degree of agreement between the expert raters, we used the mean of the raters' scores for further analysis.

Primary outcome

Participant OGRS scores on the retention day were not significantly affected by group assignment (p=0.59). The mean retention OGRS score in the simulated patient death group was 29.4 versus 29.4 in the control group (95% CI: 27.0, 31.8, and 26.8, 32.0, p=0.87). The retention OGRS score was only predicted by the participants' initial score (p=0.004, partial eta squared=0.20). The level of training did not affect retention OGRS scores (p=0.12), nor did specialty (p=0.09) (Appendix B: ANCOVA results on between-subjects effects for OGRS score). Table 3 shows the OGRS scores of participants in the study, including their initial scores and retention scores for both the simulated patient death and control groups.

**Table 3:** OGRS scores The possible range of scores is 6-42. OGRS - Ottawa Global Rating Scale

Group	Timepoint	25th	Median	75th
Death	Initial	22.56	26.00	30.50
	Retention	26.00	28.75	32.38
Survival	Initial	23.94	28.00	30.88
	Retention	26.88	29.75	33.75

Secondary outcomes

Task-Specific Checklist (Appendix C)

Checklist scores were not significantly affected by group assignment (p=0.88), with a mean retention score in the simulated patient death group of 11.8 versus 12.5 in the survival group (95% CI: 10.5, 13.0 and 11.3, 13.7, p=0.69, respectively). Level of training (p=0.08), specialty (p=0.13), and initial technical skills score (p=0.36) did not significantly affect retention checklist scores. Details are provided in Appendix B: ANCOVA results on between-subjects effects for checklist score.

Cognitive Appraisal

**Figure 2:**
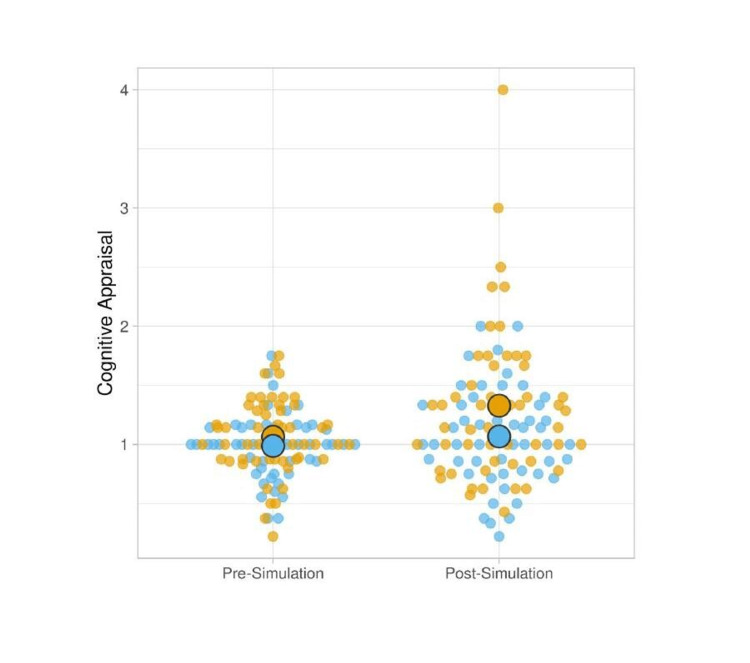
Pre- and post-simulation cognitive appraisal scores for control and death scenarios A ratio above 1 represents an appraisal as a "threat", and a ratio equal to or below 1 represents an appraisal as a "challenge". Blue dots represent participants in the scenario without simulated patient death; yellow dots represent participants in the scenario with simulated patient death. Large circles represent mean values.

We calculated the average cognitive appraisal ratio in the death and survival scenarios at each time point (Figure 2). The only significant difference from a score of 1.0 was in the post-simulation time point in the death scenario, which had an average ratio of 1.33 (95% CI: 1.14, 1.51, p=0.001), indicating that the simulated patient death group generally perceived the simulation scenario as a "threat" rather than a "challenge". The pre-test cognitive appraisal for the death scenario and both time points for the control scenario were not significantly different from 1 (Appendix B: cognitive appraisal one-sample t-test results).

State-Trait Anxiety Inventory

**Figure 3:**
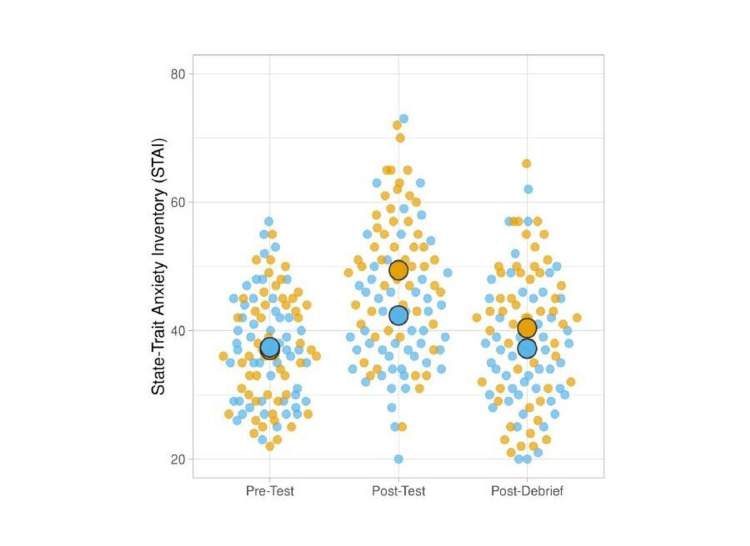
STAI at each time point for the survival (control) and death scenarios Blue dots represent participants in the scenario without simulated patient death; yellow dots represent participants in the scenario with simulated patient death. Large circles represent mean values. STAI - State-Trait Anxiety Inventory

For our secondary outcome measures, we pooled data from both the initial and retention phases and separated it by scenario type (simulated patient death or survival). Participants' anxiety scores at the second time point (immediately after the scenario) and the third time point (after the debriefing) were higher following the death scenario (B=5.466, 95% CI: 2.658, 8.275, p<0.001) and were higher on the initial day versus the retention day (B=3.792, 95% CI: 0.981, 6.604, p=0.008), regardless of which scenario was performed on each day. At the second time point, the mean STAI score for the simulated patient death scenario was significantly different from that of the control (mean difference [MD]: 7.4, 95% CI: 3.9, 10.9, p<0.001; see Figure 3). The difference between scenarios was also significant after the debriefing (MD: 3.7, 95% CI: 0.1, 7.3, p=0.04). In both scenarios, self-reported anxiety increased between the baseline and the post-scenario time point, then decreased again between the post-scenario time point and the post-debriefing time point (Appendix B: State-Trait Anxiety Inventory [STAI] scores).

Cortisol

There was no significant difference in cortisol concentration related to scenario type (B=-0.014, 95% CI: -0.995, 0.968, p=0.978) or day of the test (B=0.009, 95% CI: -0.972, 0.991, p=0.985). Cortisol concentrations varied greatly between participants and were not normally distributed. The simulated patient death scenario induced statistically significant changes to cortisol over time (p=0.004), and this was also true for the control scenario (p<0.001). However, we could not consider the between-subjects variable of scenario type and the within subjects variable of time in the same analysis using non-parametric tests. Table 4 shows the 25th, median, and 75th percentile cortisol concentrations at each time point for both scenarios.

**Table 4:** Cortisol concentrations in nmol.L-1

Scenario	Timepoint	25th	Median	75th
Death	Arrival	2.19	4.24	6.40
	Pre-scenario	2.42	4.46	6.09
	Post-scenario	3.22	5.11	7.28
	Pre-debrief	2.58	4.35	6.96
	Post-debrief	2.41	4.35	6.65
Survival	Arrival	2.89	4.94	6.82
	Pre-scenario	2.93	4.66	6.85
	Post-scenario	3.40	4.89	8.26
	Pre-debrief	2.84	3.82	6.50
	Post-debrief	2.59	4.02	6.06

Positive and Negative Emotions

Positive emotions were not significantly affected by scenario type (B=-0.789, 95% CI: -2.658, 1.079, p=0.408) or the day of the test (B=0.559, 95% CI: -1.310, 2.427, p=0.558). Negative emotions were found to be significantly higher following the simulated patient death scenario (B=2.059, 95% CI: 0.583, 3.535, p=0.006) and were not affected by the day of the test (B=0.711, 95% CI: -0.765, 2.187, p=0.345). Table 5 shows the 25th, median, and 75th percentile scores for positive and negative affect scales for both scenarios.

**Table 5:** Positive and negative affect scale The positive scale maximum is 50; the negative scale maximum is 50.

Scenario	Scale	25th	Median	75th
Death	Positive	30	33	37
	Negative	12	14	18
Survival	Positive	29	34	38
	Negative	11	13	15

## Discussion

In our study, simulated patient death during simulated-based education did not affect the learning and retention of either CRM skills or technical skills. Our exploratory secondary analyses suggest that simulated patient death may lead to greater levels of short-term anxiety, stress, and negative emotions in learners.

Lazarus's model of stress suggests that performance under a stressful condition depends on how a person appraises a perceived demand in light of the perceived resources available to address it [25,29]. The demand refers to what the person has to do to address a situation. The resources refer to personal abilities and the environmental supports available. If they perceive resources to be insufficient for the demands, the situation is perceived as a threat and can result in a lower performance with "distress," a negative psychological and physiological response that can include affective states like anxiety and increases in cortisol levels [22,29]. Conversely, if the individual determines that the resources available are sufficient to meet the demands, the situation is seen as a challenge and can result in a higher performance with "eustress," a positive state [22,29]. Our cognitive appraisal results indicate that when participants were confronted with simulated patient death, they were more likely to see the scenario as a "threat" for which they were insufficiently prepared or felt insufficiently supported, in contrast with recent work by Weiss and colleagues in which they concluded that the unexpected death of the manikin does not affect a participant's self-efficacy [30]. Weiss and colleagues focused on the expectedness of death rather than death per se: the authors demonstrated that if the manikin "dies," the participant's self-efficacy is similar whether they had been warned or not. Our study shows that unexpected and unexplained manikin death induces feelings of unpreparedness in participants compared to those who managed a similar scenario in which the manikin survived. The unexpected nature of the death may have suggested to participants that there was a detail that they missed or something they failed to consider, meaning that in their post-scenario appraisal, they considered their resources to be insufficient to address the demands of the scenario. This suggests that the introduction of death without any clear cause damages trainees' perceptions of their preparedness. This hypothesis could be explored in future studies.

Previous RCTs in this area have found conflicting results: one study found that simulated patient death was harmful to participants and worsened their retention of skills [9], while another found that it was not harmful but did not affect the retention of skills or knowledge [6]. However, the latter study included a very small number of participants, and it is possible this risked a type 2 error, missing potential harm to participants. A further difference between our study and the previous literature is that both previous RCTs included only medical students, while we included post-graduate trainees, who have an increased level of responsibility for patient care and outcomes, and so may react differently to a negative outcome. Recent work to develop a theoretical framework for simulated death indicated that when the manikin dies, participants are primarily concerned with determining what they did wrong in the simulation [31]. If the manikin always dies regardless of the participants' actions, this death has little educational value (when death is not the educational goal), as supported by our results. As shown in this study, the death of a simulated patient can lead to learners experiencing greater stress, anxiety, and negative emotions without any resulting educational benefits.

Considering that participants respond to simulated death by trying to determine what they did wrong [31], participants may believe that manikin deaths are always caused by their actions. This is supported by Weiss's finding that participants' self-efficacy was similar following a simulated death regardless of whether they were warned about the death [30]. The death of the manikin, then, may cause participants to feel that they were unprepared or unsupported and to become anxious, as they consider the death to be a reflection of their performance. This may be especially prominent in scenarios like ours, where death seemed like a very unlikely outcome, and so for the participant to cause the manikin's death, in their thinking, may have meant that they thought they missed a very important detail or made a glaring mistake. The varying degrees to which simulated patient death affects participants' short-term anxiety, stress, and negative emotions of learners observed in the literature may be connected to just how unexpected the death was in each scenario. If a simulated patient dies during a high-risk procedure or when they have many complications, the participant may perceive this death as less of an indictment of their preparedness than the death of a healthy simulated patient in a low-risk scenario.

Strengths and weaknesses

By focusing on CRM skills (i.e., non-technical skills such as communication, leadership, decision-making, and situational awareness) as our primary objective, we chose a skill set that is applicable to a range of acute and non-acute care disciplines. Our investigation of the effects of emotion and stress on learning, as well as skill retention, has implications for education outside our chosen stressor: the findings are, therefore, generalizable across many different scenarios. Our study has a number of limitations. In choosing to study the effects of simulated patient death, we are unable to comment on the use of manikin death as a teaching tool in response to participant action/inaction. This would be a different research question. It is possible that allowing the simulated patient to "survive" despite harmful or inadequate medical management would have negative educational value. Although we tried to include as broad a group of learners as possible, the majority of our participants were part of acute care teams with exposure to real-life cardiac arrest management. Given that the rate of acute resuscitation survival after adult in-hospital cardiac arrest is estimated at less than 55%, many trainees may have already been exposed to our chosen stressor [1].

The majority of our candidates were certified for advanced cardiovascular life support (ACLS) and part of emergency response teams for cardiac arrest. Our findings are, therefore, unlikely to be generalizable to more junior learners, such as medical students or those with a background in a non-acute care specialty.

Lastly, we did not specifically debrief participants in the intervention group on the outcome of manikin death. Future studies may explore the effect of simulated patient death and debriefing to unlink patient outcomes and performance. Healthcare professionals can perform perfectly, and the patient still dies, and they can perform poorly, and the patient survives. Effective debriefing following a simulated death is important to participants [31-33] and warrants further study.

## Conclusions

Our randomized controlled study showed that unexpected death during simulated cardiac arrest did not affect learning and retention of non-technical or technical CRM skills but led to greater levels of short-term anxiety, stress, and negative emotions among learners. These findings have a practical impact on simulation scenario design in the context of cardiac arrest training. Our detailed exploration of secondary end-points highlights the complex interplay between stress and emotions with respect to learning in the context of critical, life-saving skills.

## Appendices

Appendix A: Simulation Scenarios

Stem 1: Unexpected Death-Pulseless Electrical Activity-Ventricular Fbrillation-Asystole (DEATH-PEA-VF-ASYSTOLE)

Briefing

You are the on-call resident in a community hospital. You are called by a nurse to see a 21-year-old patient on a normal ward who has been admitted with an infected dog bite and cellulitis. He is being treated with intravenous antibiotics. You have been asked to urgently assess him due to the increasing agitation and complaints of breathlessness. The nurse is in the patient\s room and has called a nearby respiratory therapist (RT) for assistance.

Opening statement (T=0) by a nurse: "Thank you for coming so quickly. He's become a bit agitated and complains of feeling unwell minutes after I started the antibiotic. I'd like for you to assess him."

**Table 6:** Clinical setting at scenario start ThepPatient sitting in a normal ward bed. No monitoring is attached. No oxygen. IV-access in place on the right hand. ABC - airway, breathing, and circulation; ABG - arterial blood gas; BP - blood pressure; Cloxacillin - cloxacillin is an antibiotic medication used to treat bacterial infections; EtCO2 - end-tidal carbon dioxide; Epi-pen - epinephrine auto-injector; HR - heart rate; IM - intramuscular; IV - intravenous; RN - registered nurse; RR - respiratory rate; RT - respiratory therapist; SpO2 - peripheral oxygen saturation

Time	Actions	Manikin	Confederates	Information that MAY be provided to the participant if asked
T0	Expected to (delegate): evaluate ABC’s; recognize shock; recognize respiratory distress; give oxygen; administer IV fluids; apply monitor; give epinephrine IM	At T0: BP 60/30, HR 120, RR 28, SpO2 89% NO ETCO2 monitor, bilateral wheezes, swollen tongue, pupil size (if requested): small, equally reactive P1: "I am itchy all over" (at T=2:00) P2: "I can't breathe" (at T=2.30)	Nurse (as candidate enters room): "Thank you for coming to see this patient…" Antibiotic cloxacillin 500mg IV. Nurse (in response to P1): "Have you seen this rash?" RT (in response to P2): "His tongue seems to be swollen!" Nurse/RT brings cardiac arrest cart into room when requested	Patient responses to questions from participant: "I don’t feel great", "I don’t feel well", "I can't remember", "I don’t know". Patient's history: no known past medical history, no known allergies, no known home medications, only med given recently is the antibiotic cloxacillin 500mg IV. Patient's appearance: not swollen/no rash initially (only with cues noted), no mottling, normal breath sounds and heart sounds. Equipment and personnel: the only drugs available in the room are those in the crash cart (no epi-pen, etc). If asked for additional personnel, the code team can be called or is on their way. No other nurses or RTs are available currently. RN or RT can call for extra help, a code blue, or other investigations if asked (may use the phone in the room). RT can do ABG. RN can draw bloodwork If the participant asks where the code blue team is, the RN will "call them again" with a phone in the room, and then say: "They are just finishing up with another code blue situation and will be here in ten minutes"
T1				
T2				
T3	Expected to (delegate): recognize PEA; verbalize causes; administer drugs; deliver effective CPR; appropriate airway management	At T3: ECG sinus rhythm, BP 0/0, HR160 bpm, RR 0, SpO2 -	Cue: HR increase/disappearance of SoO2 Nurse: "I think he is not responding anymore". RT: "Is he still breathing?"	
T4				
T5				
T6	Expected to (delegate): recognize VF; appropriate defibrillation; administer drugs; continue high-quality CPR	At T6: ECG ventricular fibrillation, BP 0/0, RR 0, SpO2 -	If unrecognized at T=8 Cue: Overhead code blue call: Code Blue, Medical Ward, Room 1 Nurse: "Look, the EKG changed!"	
T7				
T8				
T9				
At T=10 min video recording is stopped. Scenario will continue for three additional minutes				
T10	Expected to (delegate): recognize asystole; continue high-quality CPR	Death: ECG asystole - at T=10.30, BP 0/0, RR 0, SpO2 - EtC	T=11 min Cue #1: RT: "What do you think happened?" T=12 min Cue #2: "He's only 21, I can't believe he arrested!"	
T11				
T12				
At T= 13: End of scenario cue: "End of scenario. Please proceed to the debriefing room." ENSURE THAT 30 MINUTE STOPWATCH IS STARTED NOW FOR CORTISOL SAMPLING				

Stem 2: Unexpected Death-Ventricular Fibrillation-Pulseless Electrical Activity-Asystole (DEATH-VF-PEA-ASYSTOLE)

Briefing

You are the on-call resident in a community hospital. You are called to a regular ward by a nurse to see a 21-year-old patient. He collapsed during a soccer game and sustained an isolated ankle fracture. He started to feel unwell a few minutes ago and has become slightly agitated.  The nurse is in the patient's room and has called a nearby RT for assistance.

Opening statement (T=0) by the nurse: "Thank you for coming to see this patient. I have a 21-year-old man in here. He is not feeling well and started to get agitated minutes after I gave him morphine for his pain."

**Table 7:** Clinical setting at scenario start The patient is sitting in a normal ward bed. No monitoring is attached. No oxygen. IV-access in place on the right hand. ABG: arterial blood gas; BP - blood pressure; CPR - cardiopulmonary resuscitation; DB1 - designated confederate 1; ECG - electrocardiogram; EtCO2 - end-tidal carbon dioxide; HR - heart rate; IV - intravenous; PEA - pulseless electrical activity; RN - registered nurse; RT -  respiratory therapist; SpO2 - peripheral oxygen saturation; VF - ventricular fibrillation

Time	Actions	Manikin	Confederates	Information that MAY be provided to the participant if asked
T0	Expected to (delegate): evaluate ABC’s; recognize shock; recognize respiratory distress; give oxygen; administer IV fluids; apply monitor; give epinephrine IM	At T0: BP 60/30, HR 120, RR 28, SpO2 89% NO ETCO2 MONITOR, bilateral wheezes, swollen tongue, pupil size (if requested): small, equally reactive. P1: "I am itchy all over" (at T=2:00) P2: "I can’t breathe" (at T=2.30)	Nurse (as candidate enters room): "Thank you for coming to see this patient…" Morphine dose 10 mg IV over 15 minutes. Nurse (in response to P1): "Have you seen this rash?" RT [DB1] (in response to P2): "His tongue seems to be swollen!" Nurse/RT brings cardiac arrest cart into room when requested	Patient responses to questions from participant: "I don't feel great", "I don't feel well", "I can't remember", "I don't know". Patient's history: no known past medical history, no known allergies. The patient has been "cleared" by the trauma service. Only med given recently is morphine 10 mg IV 10 minutes ago. Patient's appearance: not swollen/no rash initially (only with cues noted), no mottling, normal breath sounds and heart sounds. Equipment and personnel: the only drugs available in the room are those in the crash cart (no epi-pen, etc). If asked for additional personnel, the code team can be called or is on their way. No other nurses or RTs are available currently. RN or RT can call for extra help, a code blue or other investigations if asked (may use the phone in the room). RT can do ABG. RN can draw bloodwork If the participant asks where the code blue team is, the RN will "call them again" with a phone in the room, and then say "They are just finishing up with another code blue situation and will be here in ten minutes".
T1				
T2				
T3	Expected to (delegate): recognize VF; appropriate defibrillation; administer drugs; continue high-quality CPR	At T3: ECG ventricular fibrillation, BP 0/0, HR 160, RR 0, SpO2 - EtCO2 0	CUE: HR increase/disappearance of SoO2 Nurse: "I think he is not responding anymore". RT: "Is he still breathing?"	
T4				
T5				
T6	Expected to (delegate): recognize PEA; verbalize causes; administer drugs; deliver effective CPR; appropriate airway management	At T6: ECG sinus rhythm, HR 100 bpm, BP 0/0, RR 0, SpO2 - EtCO2 0	If unrecognized at T=8 Cue: Overhead code blue call (if code team previously requested). Nurse: "Look, the ECG changed!"	
T7				
T8				
T9				
At T=10 min video recording is stopped. Scenario will continue for 3 additional minutes				
T10	Expected to (delegate): recognize asystole; continue high-quality CPR	Death: ECG Asystole – at T=10.30 BP 0/0 RR 0 SpO2 - EtCO2 –	T= 11 min Cue #1: RT: "What do you think happened?" T= 12 min Cue #2: "He's only 21, I can't believe he arrested!"	
T11				
T12				
At T= 13: End of scenario cue: "End of scenario. Please proceed to the debriefing room." TEAM WILL ENSURE THAT 30 MINUTE STOPWATCH IS STARTED NOW FOR CORTISOL SAMPLING				

Stem 1: Survival-Pulseless Electrical Activity-Ventricular Fibrillation-Return of Spontaneous Circulation (SURVIVAL-PEA-VF-ROSC)

Briefing

You are the on-call resident in a community hospital. You are called by a nurse to see a 21-year-old patient on a normal ward who has been admitted with an infected dog bite and cellulitis. He is being treated with intravenous antibiotics. You have been asked to urgently assess him due to the increasing agitation and complaints of breathlessness. The nurse is in the patient’s room and has called a nearby RT for assistance.

Opening statement (T=0) by the nurse: "Thank you for coming so quickly. He's become a bit agitated and complains of feeling unwell minutes after I started the antibiotic. I’d like for you to assess him."

**Table 8:** Clinical setting at scenario start The patient sitting in a normal ward bed. No monitoring is attached. No oxygen. IV-access in place on the right hand. ABG - arterial blood gas; BP - blood pressure; CPR - cardiopulmonary resuscitation; ECG - electrocardiogram; EtCO2 - end-tidal carbon dioxide; HR - heart rate; IV - intravenous; PEA - pulseless electrical activity; ROSC - return of spontaneous circulation; RR - respiratory rate; RT - respiratory therapist; SO2 - oxygen saturation; SpO2 - peripheral oxygen saturation; VF - ventricular fibrillation

Time	Actions	Manikin		Confederates	Information that MAY be provided to the participant if asked
T0	Expected to (delegate): evaluate ABC’s; recognize shock; recognize respiratory distress; give oxygen; administer IV fluids; apply monitor; give epinephrine IM	At T0: BP 60/30, HR 120, RR 28, SpO2 89% NO ETCO2 monitor, bilateral wheezes, swollen tongue, pupil size (if requested): small, equally reactive. T=2 P1: "I am itchy all over" T= 2:30 P2: "I can't breathe"		Nurse (as candidate enters room): "Thank you for coming to see this patient…" Nurse (in response to P1): "Have you seen this rash?" RT (in response to P2): "His tongue seems to be swollen!" Nurse/RT brings cardiac arrest cart into room when requested.	Patient responses to questions from participant: "I don’t feel great", "I don’t feel well", "I can’t remember", "I don't know". Patient's history: no known past medical history, no known allergies, no known home medications. Only med given recently is the antibiotic Cloxacillin 500 mg IV. Patient's appearance: not swollen/no rash initially (only with cues noted), no mottling, normal breath sounds and heart sounds. Equipment and personnel: the only drugs available in the room are those in the crash cart (no epi-pen, etc). If asked for additional personnel, the code team can be called, or is on their way. No other nurses or RTs are available currently. RN or RT can call for extra help, a code blue or other investigations if asked (may use the phone in the room). RT can do ABG and RN can draw bloodwork If the participant asks where the code blue team is, the RN will "call them again" with a phone in the room, and then say "They are just finishing up with another code blue situation and will be here in ten minutes".
T1					
T2					
T3	Expected to (delegate): recognise PEA; verbalize causes; administer drugs; deliver effective CPR; appropriate airway management	At T3: ECG sinus rhythm, HR 160 bpm, BP 0/0, RR 0 SpO2 -		Cue: HR increase/disappearance of SoO2 Nurse: "I think he is not responding anymore". RT: "Is he still breathing?"	
T4					
T5					
T6	Expected to (delegate): recognise VF; appropriate defibrillation; administer drugs; continue high-quality CPR	At T6: ECG ventricular fibrillation, BP 0/0, RR 0, SpO2 -		If unrecognized at T=8 Cue: overhead code blue call: code blue, medical ward, room 1. Nurse: "Look, the ECG changed!"	
T7					
T8					
T9					
At T=10 min video recording is stopped. Scenario will continue for 3 additional minutes					
T10	Expected to (delegate): recognise ROSC; appropriate post-resuscitation care	Survive at T=10.5 minutes: ECG sinus rhythm, BP 90/60, HF 90, RR 10, SpO2 95	Cue: return of spO2 (saturation monitor becomes audible). Nurse: "I think he is back". If candidate fails to confirm presence of pulse, confederate will say: "I can definitely feel a pulse".		
T11					
T12					
At T= 13: End of scenario cue: "End of scenario. Please proceed to the debriefing room." ENSURE THAT 30 MINUTE STOPWATCH IS STARTED NOW FOR CORTISOL SAMPLING					

Stem 2: Survival-Ventricular Fibrillation-Pulseless Electrical Activity-Return of Spontaneous Circulation (SURVIVAL-VF-PEA-ROSC)

Briefing

You are the on-call resident in a community hospital. You are called to a regular ward by a nurse to see a 21-year-old patient. He collapsed during a soccer game and sustained an isolated ankle fracture. He started to feel unwell a few minutes ago and has become slightly agitated.  The nurse is in the patient's room and has called a nearby RT for assistance.

Opening statement (T=0) by the nurse: "Thank you for coming to see this patient. I have a 21-year-old man in here. He is not feeling well and started to get agitated minutes after I gave him morphine for his pain."

**Table 9:** Clinical setting at scenario start The patient is sitting in a normal ward bed. No monitoring is attached. No oxygen. IV-access in place on the right hand. ABC - airway, breathing, circulation; BP - blood pressure; CPR - cardiopulmonary resuscitation; ECG - electrocardiogram; EtCO2 - end-tidal carbon dioxide; HR - heart rate; IV - intravenous; PEA - pulseless electrical activity; RN - registered nurse; RT - respiratory therapist; ROSC - return of spontaneous circulation; SpO2 - peripheral capillary oxygen saturation; VF - ventricular fibrillation

Time	Actions	Manikin	Confederates	Information that MAY be provided to the participant if asked
T0	Expected to (delegate): evaluate ABC's; recognize shock; recognize respiratory distress; give oxygen; administer IV fluids; apply monitor; give epinephrine IM	At T0: BP 60/30, HR 120, RR 28, SpO2 89%, No ETCO2 monitor, bilateral wheezes, swollen tongue, pupil size (if requested): small, equally reactive. P1: "I am itchy all over" (at T=2:00). P2: "I can't breathe" (at T=2.30)	Nurse (as candidate enters room): "Thank you for coming to see this patient…" Nurse (in response to P1): "Have you seen this rash?" RT (in response to P2): "His tongue seems to be swollen!" Nurse/RT brings cardiac arrest cart into room when requested.	Patient responses to questions from participant :"I don’t feel great", "I don't feel well", "I can't remember", "I don't know". Patient's history: no known past medical history, no known allergies. The patient has been ‘"cleared" by the trauma service. Only med given recently is morphine 10 mg IV 10 minutes ago. Patient's appearance: not swollen/no rash initially (only with cues noted), no mottling, normal breath sounds and heart sounds. Equipment and personnel: the only drugs available in the room are those in the crash cart (no epi-pen, etc). If asked for additional personnel, the code team can be called, or is on their way. No other nurses or RTs are available currently. RN or RT can call for extra help, a code blue or other investigations if asked (may use the phone in the room). RT can do ABG and RN can draw bloodwork If the participant asks where the code blue team is, the RN will "call them again" with a phone in the room, and then say "They are just finishing up with another code blue situation and will be here in ten minutes".
T1				
T2				
T3	Expected to (delegate): recognize VF; appropriate defibrillation; administer drugs; continue high-quality CPR	At T3: ECG ventricular fibrillation, BP 0/0, HR 160, RR 0, SpO2 -	Cue: HR increase/disappearance of SoO2 Nurse: "I think he is not responding anymore". RT: "Is he still breathing?"	
T4				
T5				
T6	Expected to (delegate): recognize PEA; verbalize causes; administer drugs; deliver effective CPR; appropriate airway management	At T6: ECG sinus rhythm, HR 100 bpm, BP 0/0, RR 0, SpO2 - EtCO2 0	If unrecognized at T=8 Cue: overhead code blue call (if code team previously requested). Nurse: "Look, the ECG changed!"	
T7				
T8				
T9				
At T=10 min video recording is stopped. Scenario will continue for 3 additional minutes				
T10	Expected to (delegate): recognize ROSC; appropriate post-resuscitation care	Survive: ECG sinus rhythm, BP 90/60, HF 90, RR 10, SpO2 95, EtCO2 35	Cue: return of spO2 (saturation monitor becomes audible). Nurse: "I think he is back". If candidate fails to confirm presence of pulse, confederate will say: "I can definitely feel a pulse".	
T11				
T12				
At T= 13: End of scenario cue: "End of scenario. Please proceed to the debriefing room." TEAM WILL ENSURE THAT 30 MINUTE STOPWATCH IS STARTED NOW FOR CORTISOL SAMPLING				

Appendix B: Tables

**Table 10:** ANCOVA results on between-subjects effects for OGRS score OGRS_Retention: dependent variable OGRS_Initial: this variable represents the participants' initial OGRS scores, which were collected before the simulation exercise. Day_1_Scenario: this variable represents the scenario the participant experienced during their first simulation exercise, either death or survival. Level_of_Training: this variable represents the participants' level of training, such as intern, resident, or attending physician. Specialty: this variable represents the participants' medical specialty, such as emergency medicine, anesthesia, or surgery. Day_1_Scenario * Specialty: this interaction term represents the combined effect of the Day_1_Scenario and Specialty variables on the retention OGRS scores. Day_1_Scenario * Level_of_Training: this interaction term represents the combined effect of the Day_1_Scenario and Level_of_Training variables on the retention OGRS scores. Specialty * Level_of_Training: this interaction term represents the combined effect of the Specialty and Level_of_Training variables on the retention OGRS scores. ANCOVA - analysis of covariance; OGRS - Ottawa Global Rating Scale

Source	Type III sum of squares	df	Mean square	F	Sig.
Corrected model	421.169a	6	70.195	2.645	0.03
Intercept	315.298	1	315.298	11.88	0.001
OGRS_Initial	252.185	1	252.185	9.502	0.004
Day_1_Scenario	8.043	1	8.043	0.303	0.585
Specialty	79.458	1	79.458	2.994	0.091
Level_of_Training	67.554	1	67.554	2.545	0.119
Day_1_Scenario * Specialty	0	0	.	.	.
Day_1_Scenario * Level_of_Training	2.594	1	2.594	0.098	0.756
Specialty * Level_of_Training	104.34	1	104.34	3.931	0.054
Day_1_Scenario * Specialty * Level_of_Training	0	0	.	.	.
Error	1035.044	39	26.54		
Total	41178.563	46			
Corrected total	1456.213	45			

**Table 11:** ANCOVA results on between-subjects effects for checklist score Day_1_Scenario: the scenario the participant experienced during their first session, either death or survival. Tests of Between-Subjects Effects Technical_Retention: dependent variable Day_1_Scenario: this variable indicates which scenario the participant experienced during their first session (either death or survival). Level_of_Training: this variable indicates the level of training of the participant (e.g., resident, fellow, attending). Specialty: this variable indicates the medical specialty of the participant. Day_1_Scenario * Specialty: this interaction term indicates the combined effect of the scenario and the participant's specialty on their retention scores. Day_1_Scenario * Level_of_Training: this interaction term indicates the combined effect of the scenario and the participant's level of training on their retention scores. Specialty * Level_of_Training: this interaction term indicates the combined effect of the participant's specialty and level of training on their retention scores. ANCOVA - analysis of covariance

Source	Type III sum of squares	df	Mean square	F	Sig.
Corrected model	62.705a	6	10.451	1.313	0.274
Intercept	281.263	1	281.263	35.34	0
Technical_Initial	6.78	1	6.78	0.852	0.362
Day_1_Scenario	0.19	1	0.19	0.024	0.878
Specialty	19.497	1	19.497	2.45	0.126
Level_of_Training	26.423	1	26.423	3.32	0.076
Day_1_Scenario * Specialty	0	0	.	.	.
Day_1_Scenario * Level_of_Training	4.08	1	4.08	0.513	0.478
Specialty * Level_of_Training	42.671	1	42.671	5.362	0.026
Day_1_Scenario * Specialty * Level_of_Training	0	0	.	.	.
Error	310.388	39	7.959		
Total	7141.875	46			
Corrected total	373.092	45			

**Table 12:** Cognitive appraisal one-sample t-test results

	Mean	95% CI	p
Death pre	1.06	0.98 - 1.15	0.15
Death post	1.33	1.14 - 1.51	0.001
Survival pre	0.99	0.91 - 1.06	0.76
Survival post	1.07	0.95 - 1.18	0.24

**Table 13:** State-Trait Anxiety Inventory (STAI) scores

	Mean	Standard deviation	Range
Death pre	36.7	8.4	22 - 55
Death post	49.5	10.9	25 - 72
Death post debrief	40.8	10.9	21 - 66
Survival pre	37.3	8.7	23 - 57
Survival post	42.1	10.4	20 - 73
Survival post debrief	36.5	9.2	20 - 62

Appendix C: State-Trait Anxiety Inventory 

SELF-EVALUATION QUESTIONNAIRE STAI Form Y-1 and Y-2

Initials____________________________ Number of Randomization_________________________

Date__________________________

Directions:

A number of statements which people have used to describe themselves are given below. Read each statement and then circle the appropriate number to the right of the statement to indicate how you feel right now, that is, at this moment. There are no right or wrong answers. Do not spend too much time on any one statement but give the answer which seems to describe your present feelings best.

**Table 14:** Self-evaluation questionnaire STAI form Y-1 and Y-2 STAI - State-Trait Anxiety Inventory

		Not at all	Somewhat	Moderately so	Very much so
1	I feel calm	1	2	3	4
2	I feel secure	1	2	3	4
3	I am tense	1	2	3	4
4	I feel strained	1	2	3	4
5	I feel at ease	1	2	3	4
6	I feel upset	1	2	3	4
7	I am presently worrying over possible misfortunes	1	2	3	4
8	I feel satisfied	1	2	3	4
9	I feel frightened	1	2	3	4
10	I feel comfortable	1	2	3	4
11	I feel self-confident	1	2	3	4
12	I feel nervous	1	2	3	4
13	I am jittery	1	2	3	4
14	I feel indecisive	1	2	3	4
15	I am relaxed	1	2	3	4
16	I feel content	1	2	3	4
17	I am worried	1	2	3	4
18	I feel confused	1	2	3	4
19	I feel steady	1	2	3	4
20	I feel pleasant	1	2	3	4
21	I feel pleasant	1	2	3	4
22	I feel nervous and restless	1	2	3	4
23	I feel satisfied with myself	1	2	3	4
24	I wish I could be as happy as others seem to be	1	2	3	4
25	I feel like a failure	1	2	3	4
26	I feel rested	1	2	3	4
27	I am “calm, cool and collected”	1	2	3	4
28	I feel that difficulties are piling up so that I cannot overcome them	1	2	3	4
29	I worry too much over something that really doesn’t matter	1	2	3	4
30	I am happy	1	2	3	4
31	I have disturbing thoughts	1	2	3	4
32	I lack self-confidence	1	2	3	4
33	I feel secure	1	2	3	4
34	I make decisions easily	1	2	3	4
35	I feel inadequate	1	2	3	4
36	I am content	1	2	3	4
37	Some unimportant thought runs through my mind and bothers me	1	2	3	4
38	I take disappointments so keenly that I can’t put them out of my mind	1	2	3	4
39	I am a steady person	1	2	3	4
40	I get in a state of tension or turmoil as I think over my recent concerns and interests	1	2	3	4

Appendix C: Task-specific skills checklist

**Table 15:** Task-specific skills checklist PEA - pulseless electrical activity; ROSC - return of spontaneous circulation; VF - ventricular fibrillation

Participants initials________________ Randomization number_______________				Date__________
Critical performance steps				Done correctly
Team leader				
Ensures high-quality CPR at all times				
Assigns team member roles				
Ensures that team members perform well				
Tachycardia management				
Starts oxygen if needed, places monitor, starts IV				
Places monitor leads in proper position				
Recognizes tachycardia				
Recognized respiratory distress				
Gives appropriate initial drug therapy (epinephrine IM 0.2-0.5 mg, fluids IV)				
PEA management				
Recognizes PEA				
Verbalizes potential reversible causes of PEA/asystole (H’s and T’s)				
Administers appropriate drug(s) and doses				
Immediately resumes CPR after rhythm and pulse checks				
Appropriate airway management				
VF Management				
Recognizes VF				
Clears before ANALYZE and SHOCK				
Immediately resumes CPR after shocks				
Appropriate cycles of drug-rhythm check/shock-CPR				
Administers appropriate drug(s) and doses				
STOP TEST				
Test Results	Pass	Fail	Score_________ of 18	
Investigator				
Date				
Signature				
